# Acupuncture for Pain Management in Cancer: A Systematic Review and Meta-Analysis

**DOI:** 10.1155/2016/1720239

**Published:** 2016-02-10

**Authors:** Caiqiong Hu, Haibo Zhang, Wanyin Wu, Weiqing Yu, Yong Li, Jianping Bai, Baohua Luo, Shuping Li

**Affiliations:** ^1^The Second Clinical Medical School, Guangzhou University of Chinese Medicine, Guangzhou 510405, China; ^2^Department of Oncology, Guangdong Provincial Hospital of Chinese Medicine, Guangzhou 510120, China

## Abstract

*Objective*. To evaluate the effectiveness and safety of acupuncture for cancer-related pain.* Methods.* A systematic review of literatures published from database inception to February 2015 was conducted in eight databases. RCTs involving acupuncture for treatment of cancer-related pain were identified. Two researchers independently performed article selection, data extraction, and quality assessment of data.* Results.* 1,639 participants in twenty RCTs were analyzed. All selected RCTs were associated with high risk of bias. Meta-analysis indicated that acupuncture alone did not have superior pain-relieving effects as compared with conventional drug therapy. However, as compared with the drug therapy alone, acupuncture plus drug therapy resulted in increased pain remission rate, shorter onset time of pain relief, longer pain-free duration, and better quality of life without serious adverse effects. However, GRADE analysis revealed that the quality of all outcomes about acupuncture plus drug therapy was very low.* Conclusions.* Acupuncture plus drug therapy is more effective than conventional drug therapy alone for cancer-related pain. However, multicenter high-quality RCTs with larger sample sizes are needed to provide stronger evidence for the effectiveness of acupuncture in cancer-related pain due to the low data quality of the studies included in the current meta-analysis.

## 1. Introduction

Pain is one of the most common symptoms associated with cancer. It may be caused directly by the cancer lesion or by anticancer treatments administered to the patients. It was reported that approximately 25% newly diagnosed cancer patients, 33% patients undergoing anticancer treatments, and 75% patients with advanced cancer suffer from pain [1]. Pain is one of the symptoms cancer patients fear the most. Unrelieved pain causes discomfort in patients and greatly affects their overall quality of life [2]. Mounting evidences show that survival of cancer patients is linked to effective pain management [3]. Thus, a three-step ladder approach for cancer pain relief was highly recommended by the World Health Organization (WHO) for cancer treatment, which had shown outstanding effectiveness in alleviating cancer pain when applied appropriately. Nevertheless, studies have shown that at least 20–40% of cancer pain were not adequately relieved by application of the analgesic ladder [4, 5]. Moreover, analgesic pharmaceutical drugs are usually associated with a variety of adverse side effects, such as constipation, urinary retention, nausea, sedation, respiratory depression, myoclonus, delirium, sexual dysfunction, and hyperalgesia [6]. On the other hand, the noninvasive Complementary and Alternative Medicine (CAM) is generally considered to be relatively safe and thus is often used as an auxiliary therapy in addition to other standard pain management techniques [7].

Among a variety of CAM treatments, acupuncture is the most widely used intervention, which is used to treat many symptoms and conditions associated with cancer and adverse effects related to cancer treatments [8]. It has been shown that acupuncture is safe and minimally invasive, with very few adverse effects. In the past 20 years, many studies reported use of acupuncture for cancer pain relief. However results of these studies were not consistent. Therefore, it is still difficult for the physicians to make informed decision to include acupuncture in a cancer patients' treatment plan.

Currently, there are several systematic literature reviews [9–12] on the use of acupuncture for cancer pain management. However, the review by Lee et al. [9] included non-RCT studies. The review by Paley et al. [10] just included three randomized clinical trials (RCTs) and is now outdated. The review by Peng et al. [11] included seven RCTs but did not use meta-analysis. A more recent review published in 2013 [12] evaluated evidences from RCTs regarding use of acupuncture for cancer pain relief by meta-analysis, but only nine RCTs were included in their analysis.

Now with more and more newly published literatures, it is necessary to perform systematic review again on the use of acupuncture on cancer pain relief to update current knowledge and completely evaluate available experimental evidences in order to guide future research and practice.

## 2. Methods

### 2.1. Inclusion Criteria

The inclusion criteria were (1) study design: RCTs investigating the use of acupuncture for cancer pain relief that contain clinical data, regardless of publication status and language, (2) participants: adult patients diagnosed with any stage of cancer who experienced cancer pain, (3) intervention and control: acupuncture was used as the sole intervention or as an auxiliary therapy for other standard treatments for cancer pain, and a control group received standard treatments or placebo treatment. If acupuncture plus conventional drug therapies was compared with conventional drug therapies alone, the use of analgesic drug must be unchanged during the study period, so as to ensure the effect of acupuncture on cancer pain relief clearly, and (4) outcome measures: the primary outcome was the analgesic effect validated with a pain measurement, such as the Visual Analog Scale for Pain (VAS Pain), the Numeric Rating Scale for Pain (NRS Pain), or the McGill Pain Questionnaire. Secondary outcomes can include quality of life, patient satisfaction, frequency of hospitalization, and side effects.

### 2.2. Exclusion Criteria

The exclusion criteria were (1) animal studies, case reports, qualitative studies, descriptive surveys, and reports that were available only in abstract form, (2) trials that studied pain which cannot be clearly attributed to cancer or cancer treatment; for example, trials involve patients at a few days after surgical resection of malignant tumors, (3) trials that adopted CAM treatments that were expected to have similar effects to acupuncture (e.g., moxibustion, transcutaneous electrical nerve stimulation, point injection, laser irradiation, cupping, and tuina), (4) trials that cannot clearly evaluate the treatment efficacy of acupuncture (e.g., two different forms of acupuncture were used for different groups), and (5) outcome measures that were not relevant to cancer pain.

### 2.3. Search Strategy

#### 2.3.1. Electronic Search

Cochrane Central Register of Controlled Trials, PubMed, Embase, Web Of Science, Chinese BioMedical Literature Database (CBM), VIP Database for Chinese Technical Periodicals (VIP), China National Knowledge Infrastructure (CNKI), and Wanfang Data were searched from database inception to February 2015. No language limitations were applied. The following search terms were used as subject terms and free terms: cancer, tumor, carcinoma, neoplasms, pain, acupuncture, electroacupuncture, auriculotherapy, acupoint, and needle. EndNote software was used to manage citations obtained through the databases search.

#### 2.3.2. Manual Search

Acupuncture Research, Chinese Acupuncture and Moxibustion, Shanghai Journal of Acupuncture and Moxibustion, Journal of Chinese Integrative Medicine, and Liaoning Journal of TCM were searched from 2010 to 2015 to identify additional studies.

#### 2.3.3. Study Selection, Data Extraction, and Quality Assessment

Two researchers (Caiqiong Hu and Weiqing Yu) independently examined the articles according to the inclusion criteria and extracted data for analysis. Disagreements were resolved by discussion and consensus and mediated by a third reviewer (Haibo Zhang). The risk of bias was assessed using the Cochrane collaboration tool. The Grading of Recommendations Assessment, Development and Evaluation (GRADE) framework was applied to assess the quality of evidence for each outcome, and the results were summarized in a Summary of Findings Table using GRADEpro 3.6.1 software.

#### 2.3.4. Data Analysis

RevMan 5.3 software was used to analyze the results. Dichotomous and continuous data were presented as relative risk (RR) and mean difference (MD), respectively, with 95% confidence interval (CI). Heterogeneity among trials was identified by the *χ* ^2^ test. When heterogeneity test was acceptable (*P* > 0.1, *I* ^2^ ≤ 50%), a fixed-effects model was used for meta-analysis. When heterogeneity was significant (*P* ≤ 0.1, *I* ^2^ > 50%), it was analyzed with consideration of clinical factors, such as the type of cancer, duration of follow-up, and methodological factors, such as randomization, allocation concealment, and blinding. Subgroup analysis was performed when heterogeneity was detected significantly. A random-effects model was used to pool data if excessive statistical heterogeneity was present. Descriptive analysis was performed in case of unacceptable clinical heterogeneity. Sensitivity analysis was carried out to evaluate stability of results. When at least 9 trials were available for a meta-analysis, likelihood of publication bias was assessed by Stata 12.0 to construct funnel plots.

## 3. Results

**Figure 1:**
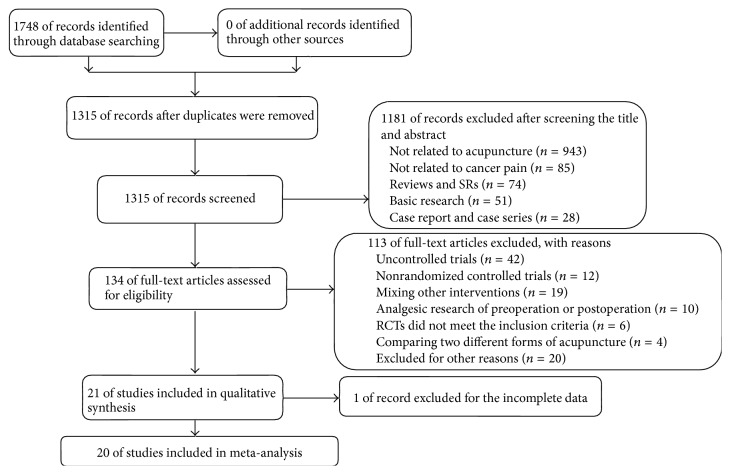
Flow chart of the publication selection process.

A total of 1,748 articles were screened, and 1,315 remained after duplicate records were deleted. Another 1,181 articles were excluded after examining title and abstract for nonclinical trials or irrelevant to cancer pain. Further full-text reading excluded 108 articles as they were non-RCTs, lack control groups, or had mixed interventions. Three [13–15] were excluded due to use of different analgesic frequency or doses in the acupuncture group, and two articles [16, 17] were excluded because of the unavailable data. Total of 21 studies were included in the final qualitative analysis, among which one article [18] was not included in the meta-analysis due to incomplete data. Finally, 1,639 participants in 20 studies were included in our meta-analysis, among which 19 trials [19–37] were published in Chinese and one [38] was published in English. Flow diagram of the publication selection process was summarized in Figure 1.

### 3.1. Study Description (Table 1)

#### 3.1.1. Participants

We included 21 studies in the final qualitative analysis, among which 20 studies were conducted in China and one study was conducted in France. Two studies were published in English and all others were published in Chinese. The types of cancer in these trials were six RCTs [20, 21, 23–26] of liver cancer, two RCTs [19, 33] of stomach cancer, one [18] of pancreatic cancer, and other RCTs of miscellaneous cancers. The largest sample size was 207 cases in a study by Tan et al. [31] while the smallest sample size was 22 cases in a pilot study by Peng [32].

#### 3.1.2. Acupuncture Interventions

The acupuncture style, needling method, number of sessions, and duration of each session all varied among trials included in this study. Most of the trials used manual acupuncture based on Traditional Chinese Medicine theory. Five studies used electroacupuncture (EA) [18, 22, 24, 34, 36], and three studies used wrist-ankle acupuncture [20, 21, 26]. One study used ear acupuncture [38], and one study used ear acupuncture and electroacupuncture (EA) [32]. One study used manual acupuncture and fire needle [19], and another study used manual acupuncture alone. For acupoint selection, most of the trials used a predetermined set of acupoints combined with a set of variable acupoints (such as Back-shu points corresponding to the anatomical Zang-Fu organs and the pain points). In addition, five studies used Ashi points in all participants [20, 21, 25, 26, 28] and one study used Eight Methods of Intelligent Turtle when choosing acupoints [23]. Furthermore, one study selected acupoints according to electrodermal response on the ear [38]. The De Qi sensation, a needling sensation perceived as numbness, soreness, or distension that is usually generated by manipulating acupuncture needles for the intended therapeutic effect, was reported in fourteen studies, but not in the other seven studies [19–21, 25, 26, 30, 38]. The number of acupuncture sessions administered ranged from 1 to 60, one session per day. The needling depth, acupuncture manipulation, and needle retention time varied among the included studies. Only one study [18] reported that the therapist was an acupuncturist with 15 years of clinical experience, while no other included studies reported the experience of the therapist.

#### 3.1.3. Controls

In seven studies, acupuncture plus conventional drug therapies was compared with conventional drug therapy alone [19, 22, 23, 31, 34, 35, 37]. Six studies tested the effects of acupuncture compared with conventional drug therapies [21, 25, 26, 29, 30, 33]. Three studies had acupuncture group, acupuncture combined with drug analgesic group, and drug analgesic group alone [20, 27, 36]. Three studies used nonpenetrating sham acupuncture controls in the control group at identical acupuncture points used for the acupuncture group [18, 32, 38]. In one study, acupuncture was compared with transdermal fentanyl [24], and in one other study, acupuncture combined with chemotherapy and drug analgesic therapy was compared with chemotherapy plus drug analgesic therapy [28].

#### 3.1.4. Outcomes

Most of the studies used pain remission rate as the primary outcomes to evaluate analgesic effectiveness. Three studies evaluated analgesic effectiveness by change in VAS score [24, 32, 38] and one study by change in NRS score [18]. For the secondary outcomes, four studies evaluated patients' quality of life, onset time of pain relief, and duration of pain relief [22, 23, 28, 37]. Two studies evaluated patients' quality of life, spiritual state of pre- and posttreatment [32, 37], and one study evaluated difference in electrical potential of the ear in pre- and posttreatment [38]. For cancer pain grading, numerical rating scale (NRS), verbal rating scale (VRS), and visual analogue scale (VAS) are more commonly used methods. Seven studies used NRS for pain assessment [11, 18, 21–23, 26, 34, 37]; seven studies used VRS [19, 27, 29, 31, 33, 35, 36]; three studies used VAS [24, 32, 38]; and two studies used NRS combined with VRS [20, 28]. Nevertheless, two studies did not describe the method by which the intensity of pain was evaluated [25, 30].

#### 3.1.5. Risk of Bias (Figure 2)

All included RCTs were associated with a high risk of bias. Of the twenty-one included RCTs, six RCTs used random-number table to generate subject ID [18, 19, 23, 24, 28, 32], four trials used visiting sequence [20, 21, 26, 37], one study used random sequence generated by computer [38], and the rest ten RCTs did not describe the randomization method for subjects. Allocation concealment was appropriately employed in four RCTs using sealed envelope [18, 23, 24, 32] and by computer [38], but the rest of the trials did not provide information about allocation concealment. Furthermore, two RCTs [32, 38] described blinding of both participants and outcome assessment, and two RCTs [18, 24] described blinding of outcome assessment. In addition, only five RCTs reported dropout or withdrawal rate [18, 24, 28, 32, 38]. Risk of other biases may exist in these analyzed trials. However, there was no sufficient information to evaluate the likelihood for the presence of other biases, or if an identified problem in the study was sufficient to introduce biases.

## 4. Meta-Analysis

### 4.1. Acupuncture versus Drug Therapy

#### 4.1.1. Response Rate for Pain Relief

**Figure 3:**
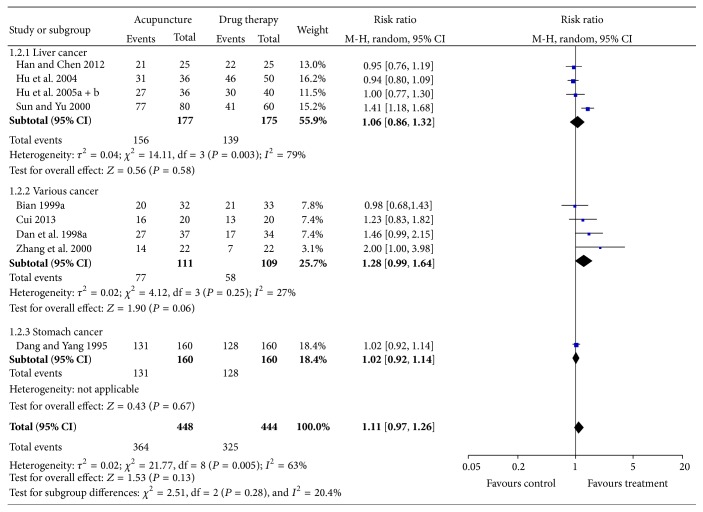
Forest plot of acupuncture for the treatment of cancer pain compared with drug therapy alone.

**Figure 4:**
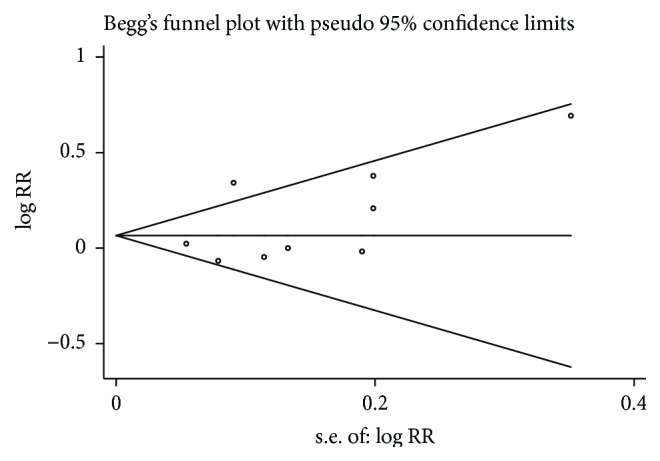
Funnel plot of acupuncture for the treatment of cancer pain compared with drug therapy alone.

Nine RCTs compared the effects of acupuncture with conventional drug therapies [20, 21, 25–27, 29, 30, 33, 36]. Meta-analysis showed significant heterogeneity (*I* ^2^ = 63%, *P* = 0.005). A random-effects model was used for statistical analysis (Figure 3). Our results showed that acupuncture does not show superior effects to drug therapy on pain relief (*n* = 892, RR = 1.11, *P* = 0.13, and 95% CI: 0.97–1.26). As different types of cancer were included in different trials, a subanalysis was performed to explore whether the heterogeneity could be partially explained by different types of cancer. Our results showed that acupuncture treatment did not improve pain relief in any specific type of cancer patients in the subgroup analysis. Moreover, significant heterogeneity was detected in the “liver cancer” subgroup (*I* ^2^ = 79%, *P* = 0.003). Since three trials used visiting sequence for sequence generation [20, 21, 26], the other one trial [25] was excluded from the sensitivity analysis, and the heterogeneity was reduced as a result (*I* ^2^ = 0%, *P* = 0.90). However, our result still failed to show superior effects of acupuncture on pain relief (RR = 0.95, 95% CI: 0.85–1.07). For publication bias, Begg's test did not suggest asymmetry in the funnel plot (*P* = 0.076), Figure 4.

### 4.2. Acupuncture Plus Drug Therapy versus Drug Therapy Alone

#### 4.2.1. Response Rate for Pain Relief

**Figure 5:**
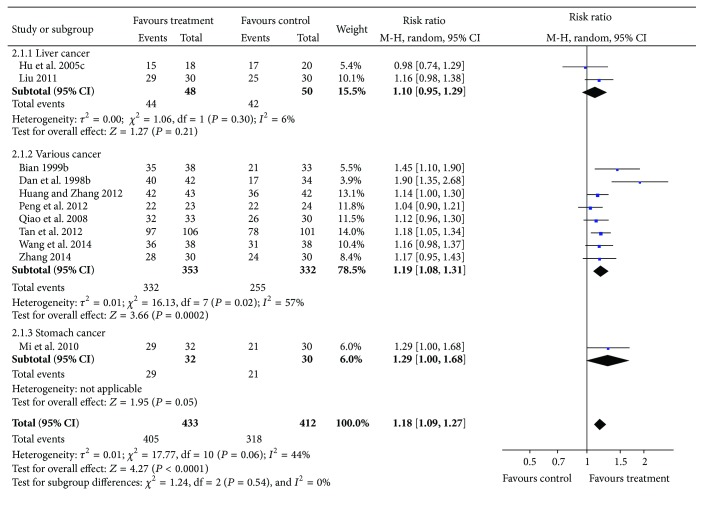
Forest plot of acupuncture plus drug therapy compared with drug therapy alone.

**Figure 6:**
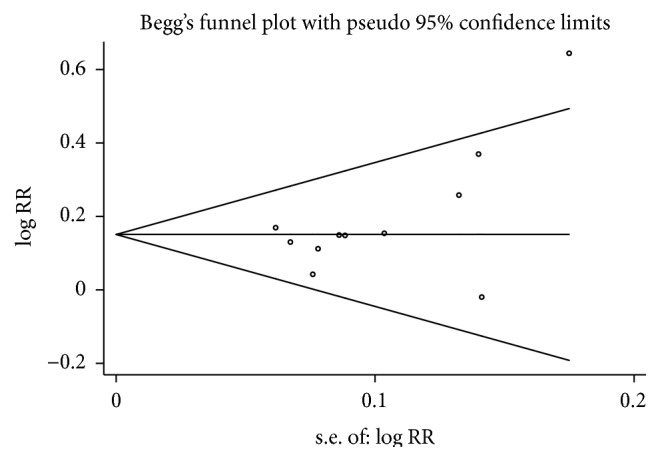
Funnel plot of acupuncture plus drug therapy compared with drug therapy alone.

Eleven RCTs compared the effects of acupuncture plus drug therapy with drug therapy alone [19, 20, 22, 23, 27, 28, 31, 34–37]. Meta-analysis showed moderate heterogeneity (*I* ^2^ = 44%, *P* = 0.06). A random-effects model was used for statistical analysis (Figure 5) and analysis showed favorable effects of acupuncture plus drug therapy on pain reduction as compared to the drug alone group (*n* = 845, RR = 1.18, *P* < 0.0001, and 95% CI: 1.09–1.27). For publication bias, Begg's test did not detect asymmetry in the funnel plot (*P* = 0.119), Figure 6.

#### 4.2.2. Onset Time of Pain Relief

**Figure 7:**
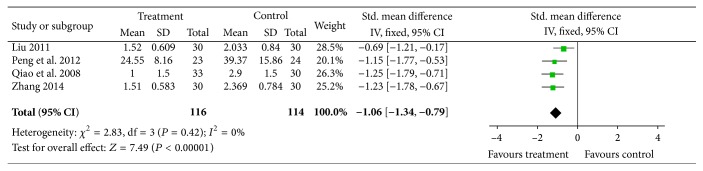
The onset time of pain relief: acupuncture plus drug therapy versus drug therapy alone.

Four trials evaluated the onset time of pain relief [23, 28, 34, 37], for which heterogeneity test was acceptable (*I* ^2^ = 0%, *P* = 0.42). Therefore, a fixed-effects model was used for statistical analysis. Our results showed that acupuncture plus drug therapy led to significantly shortened onset time of pain relief (*n* = 230, SMD = −1.06, *P* < 0.00001, and 95% CI: −1.34 ~−0.79), Figure 7.

#### 4.2.3. Duration of Pain Relief

**Figure 8:**
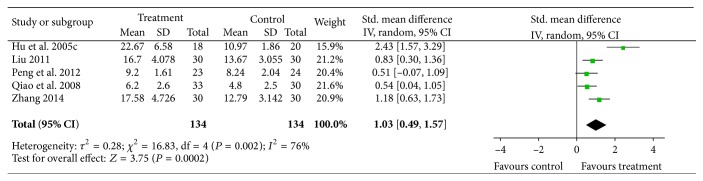
The duration time of pain relief: acupuncture plus drug therapy versus drug therapy alone.

Five trials evaluated the duration of pain relief [20, 23, 28, 34, 37], for which meta-analysis showed significant heterogeneity (*I* ^2^ = 76%, *P* = 0.002) and the heterogeneity could not be explained by clinical or methodological factors. Thus, a random-effects model was used for statistical analysis. Analysis results showed significant effects of acupuncture on prolonging the analgesic time (*n* = 268, SMD = 1.03, *P* = 0.0002, and 95% CI: 0.49–1.57), Figure 8.

#### 4.2.4. Quality of Life

**Figure 9:**
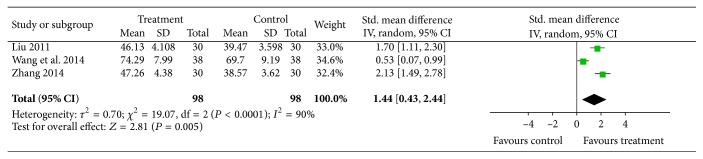
Quality of life: acupuncture plus drug therapy versus drug therapy alone.

Three trials reported the patients' quality of life in pre- and posttreatment. The study by Wang et al. [22] used Karnofsky Performance Status (KPS) for assessment while the other two trials [23, 37] used quality of life questionnaires. Meta-analysis showed significant heterogeneity (*I* ^2^ = 90%, *P* < 0.0001), and a random-effects model was used for statistical analysis. Analysis result detected significant effects of acupuncture on improving the patients' quality of life (*n* = 268, SMD = 1.03, *P* = 0.0002, and 95% CI: 0.49–1.57). As the study by Wang et al. [22] used different assessment tools, this study was excluded from the sensitivity analysis, and the heterogeneity became acceptable. However, analysis results were not affected, Figure 9.

### 4.3. Acupuncture versus Sham Acupuncture

**Figure 10:**
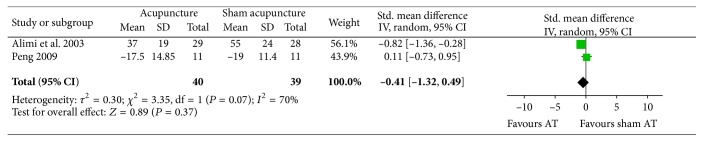
Forest plot of acupuncture compared with sham acupuncture.

Two RCTs assessed the effects of acupuncture on cancer pain as compared with sham acupuncture [32, 38]. One RCT showed significantly favorable effects of acupuncture but not the other trial. Reduction of cancer pain in the acupuncture group was not statistically significant as compared with the sham acupuncture group (*n* = 79, SMD = −0.41, *P* = 0.37, and 95% CI: −1.32–0.49; *I* ^2^ = 70%, *P* = 0.07), Figure 10.

### 4.4. Adverse Events

Of the twenty included RCTs, thirteen trials assessed adverse effects but not the others [25, 27, 29, 30, 32, 34, 36]. The main adverse reactions in the drug therapy group were nausea, vomiting, constipation, dizziness, fatigue, and urinary retention. In acupuncture therapy group, one trial [24] reported that three patients had subcutaneous bruises, which disappeared after hot compress every other day. One trial [24] reported adverse effects such as subcutaneous hemorrhage and fainting during acupuncture, with an incident rate of 8%.

## 5. Discussion

### 5.1. Summary of the Main Results

Twenty studies involving a total of 1,639 patients were included in this meta-analysis study. Sample size varied from 22 to 207 participants. The main findings were that compared with conventional drug therapy; acupuncture therapy alone did not show superior effects on cancer pain relief. Acupuncture plus drug therapy resulted in increased remission rate of pain, shorter onset time of pain relief, longer duration of analgesic time, and better quality of life. Only two RCTs compared the effects of acupuncture on cancer pain with sham acupuncture, and there was no significant difference on cancer pain reduction. However, the evidences used in this meta-analysis were insufficient to warrant a clinical recommendation due to the generally low quality of methodology of the included studies.

### 5.2. Quality of Evidence

The Cochrane Collaboration Network GRADE was used to perform a systematic review of the results (Table 2). The systematic analysis contained four outcomes in the acupuncture plus drug group and the drug alone group. Response rate for pain relief was key outcome while the onset time of pain relief, duration of pain relief, and quality of life were important secondary outcomes. GRADE profile indicated that the quality of evidence for all outcomes was very low. The low quality was primarily caused by methodological limitations, and the inconsistency could not be explained. Limited sample sizes and small overlap of the confidence intervals may cause data inaccuracy. Moreover although incomplete data was only limited to a small number of trials, all analysis showed benefits of the studied intervention, implying publication bias. Overall, these weaknesses cause reduction in the quality of data.

### 5.3. Limitations of Included Studies

#### 5.3.1. Quality of Methodology

Firstly, quality of methodology of the included studies was poor. Of the twenty included RCTs, ten RCTs did not provide detailed description of the randomization process; sixteen RCTs did not provide information about allocation concealment; only three RCTs described the blinding process, and only four RCTs reported information on trial dropout or withdrawal. In addition, no description was available to confirm that these studies were free of selective reporting bias. These different types of bias could therefore lead to false-positive results. In addition, the calculation method for sample size was not reported in any study, and thus these clinical trials may have extremely low power for statistical analysis.

#### 5.3.2. Inconsistent Interventions

Of the twenty included RCTs, there were varying experimental groups, such as acupuncture group versus drug therapy group, acupuncture plus drug therapy versus drug therapy alone, and acupuncture versus sham acupuncture. The number of acupuncture sessions, needling depth, acupuncture manipulation, and needle retention time were all different among the included studies, which might have introduced bias. In addition, most of trails did not follow up patients after acupuncture treatment, and thus the long-term analgesic effect according to the duration and frequency of acupuncture was unclear. For controls, two RCTs used sham acupuncture and one RCT used auricular seeds at acupoints that elicited no electrical response. Both studies showed significant favorable effects of acupuncture. On contrary, other trial used shallow needling at nonacupoints but did not show a positive analgesic effect. As placebo and control groups are limited, the conclusion that acupuncture is more effective than sham acupuncture is weak.

#### 5.3.3. Limited Outcomes

Most of the included trials did not describe adverse effects and prognosis. As a result, we were unable to adequately assess the effectiveness and safety of acupuncture for cancer pain relief. In addition, all included RCTs used the improvement of subjective symptoms as their study outcomes. Only three trials reported change in physical-chemical indexes in pre- and posttreatment time points. For example, the study by Dang and Yang [33] reported that content of leucine-enkephalin (LEK) in the acupuncture group increased markedly while there was no significant change in the drug therapy group. Rate of E-rosette formation was increased in the acupuncture group while it was decreased in the control group. Activity of copper-zinc superoxide dismutase (Cu/Zn-SOD) increased corresponding to increase in analgesic effects by acupuncture in the cancer patients, but the level was not as high as that in healthy volunteers. A study by Hu et al. [20] reported that the content of plasma *β* endorphin in patients with liver cancer pain was lower than healthy volunteers and liver cancer patients without pain, but the content of Substance P was not significantly different among these groups. After treatment, the content of plasma *β* endorphin in the acupuncture group increased markedly and the content of Substance P decreased markedly. Moreover, a study of Peng et al. [34] also found that the content of plasma *β* endorphin in acupuncture plus drug therapy group was significantly higher than that in the drug therapy alone group after treatment. Although it seems that acupuncture has certain therapeutic values for cancer pain, objective index to evaluate these analgesic effects is still inadequate. As physical-chemical indexes are widely used in experimental studies for the effect of acupuncture [39–45], future clinical trial may consider the use of those indexes as outcome indicators.

#### 5.3.4. Lack of Economic Data

No economic data or relative economic analysis has been reported.

### 5.4. Limitations of the Current Study

Although considerable amount of efforts was spent to retrieve RCTs and evaluate data quality, and GRADE framework was applied to assess the quality of evidence for certain outcomes, the current study still has several limitations. For example, almost all trials included in the analysis were published in Chinese journals, which may limit the scope of generalization of our findings. Moreover, in spite of extensive literature searching, we may have missed some supplementary issues, conference papers, and gray literature as these may not be available in the databases. In addition, funnel plot was not generated for some of the outcomes due to limited number of included trials. Therefore, publication bias might exist, but we failed to detect that. Finally, quality of the evidences was weak due to limited number of included trials and imperfect study design.

### 5.5. Comparison with Other Review Studies

Although there were several systematic reviews of acupuncture for cancer pain relief [9–12], only the study by Choi et al. [12] performed meta-analysis of RCTs. Therefore, it is most relevant to compare our study to the study by Choi et al. We identified ten new RCTs and updated the evidences [18, 22–24, 26, 30, 31, 34, 35, 37]. After stringent screening of the included trials and reexamination of the raw data for outcomes, we excluded two trials that were published on unqualified periodicals and one trial [16] with incomplete data. The review by Choi et al. suggested that acupuncture did not have a better effect than the drug therapy (*n* = 886, RR = 1.12, 95% CI: 0.98–1.28, and *P* = 0.09). Comparison of the acupuncture plus drug therapy and drug therapy alone detected a significant difference in favor of the combination therapy (*n* = 437, RR = 1.36, 95% CI: 1.13–1.64, and *P* = 0.003), consistent with our analysis result. Nevertheless, in addition to analyzing the response rate for pain relief, we also analyzed other parameters such as onset time of pain relief, duration of pain relief, and quality of life. Finally, we used GRADE framework to assess the quality of evidence for certain outcomes.

## 6. Conclusions

### 6.1. Implications for Clinical Practice

Results of this systematic review suggested that compared with conventional drug therapy acupuncture alone did not show superior effects on pain relief. Acupuncture plus drug therapy resulted in increased remission rate of pain, shorter onset time of pain relief, longer duration of analgesia time, and better quality of life without serious adverse effects, as compared with drug therapy alone. However, the evidences available for this systematic review are insufficient to endorse a routine use of acupuncture for cancer pain relief because of the methodological limitations of the included studies.

### 6.2. Implications for Future Research

Since the methodological quality of RCTs in the current study is relatively low, prospective, multicenter, and large-scale clinical trials with high quality are needed. We suggest that future clinical trials should be registered in the WHO International Clinical Registry Platform in advance and reported in detail according to the CONSORT [46] (Consolidated Standards for Reporting of Trials) or STRICTA [47] (Standards for Reporting Interventions in Controlled Trials of Acupuncture) guidelines. A sample size calculation should be conducted before enrollment. Randomization, allocation concealment, and blinding should be designed and carried out appropriately. In addition, duration of follow-up should be sufficient for optional long-term effectiveness and safety studies. Furthermore, reasonable placebo-control should be included in line with modern human research ethics. Finally, in addition to subjective symptom relief reported by the patients, future clinical trial can introduce more objective outcome measures such as these physical-chemical indexes. Furthermore, more attention should be paid to side effects, patient's overall well-being, and quality of life and so on. In other words, more precise, systematic, and objective outcomes should be applied to evaluate the effects of acupuncture for cancer pain based on our analysis.

## Figures and Tables

**Figure 2:**
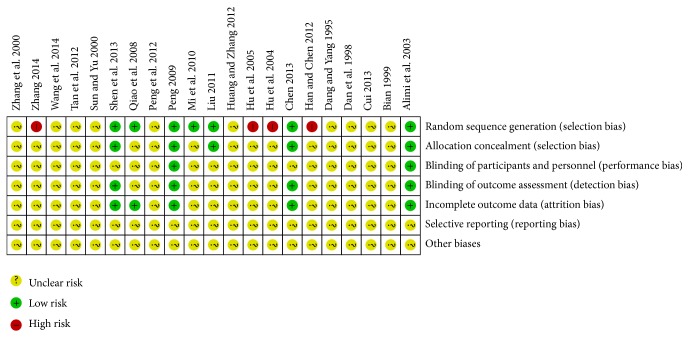
Summary of risk of bias.

**Table 1:** Characteristics of the included studies.

Study (year)	Type of cancer	Sample sizes		Interventions		Acupuncture point selection	Frequency Session duration	Main outcomes Assessment tool of pain	*P* value of main outcome
		T	C	T	C				
Chen et al. [18] (2013)	Pancreas	30	30	EA + drug	Placebo + drug (three-step analgesic ladder)	Jiaji (Ex-B2) points from T8 to T12 bilaterally	30 min qd 3 days	Pain in score NRS	*P* < 0.001

Mi et al. [19] (2010)	Stomach	32	30	Fire needle + C	Drug (three-step analgesic ladder)	Fire needle point: BL17, BL18, BL21 Manual acupoint: CV12, ST25, and ST36	30 min qod 4 weeks	Reduction of pain level VRS	*P* < 0.05

Hu et al. [20] (2005)	Liver	20 16 18	20 20 20	A: wrist-ankle AT B: wrist-ankle AT C: AT + MS Contin	Codeine 30 mg tid MS Contin MS Contin	Ashi point, primary lesion	10–12 h 10 days	Response rate (reduction of pain more than 1/2) NRS plus VRS	*P* < 0.05 *P* < 0.05 *P* > 0.05

Hu et al. [21] (2004)	Liver	36	50	Wrist-ankle AT	Drug (three-step analgesic ladder)	Ashi point, primary lesion	9–12 h, qd or qod 10 days	Response rate NRS	*P* > 0.05

Wang et al. [22] (2014)	Miscellaneous	38	38	EA + C	Drug (three-step analgesic ladder)	Jiaji points from T2 to T6 Chest pain: BL13, BL43 Limbs pain: LI11, SJ5, GB30, and GB34 Headache: GB20 Low-back pain: BL23 Abdominal pain: SP6, ST36	15 min, qod 30 days	(1) Response rate (2) KPS NRS plus VRS	*P* < 0.05

Liu [23] (2011)	Liver	30	30	AT + tramadol hc	Tramadol hc 100 mg q12 h	SP4, PC6, GB40, SJ5, SI3, BL62, LU7, KI6, LR3, and LR14	30 min qd 4 weeks	(1) Reduction of pain level (2) QOL NRS	*P* > 0.05

Shen et al. [24] (2013)	Liver	30	30	EA	Duragesic	DU20, LI11, PC6, SP6, SP10, ST36, LR3, KI3, and GB41	20 min qd 14 days	Pain in score (100 mm VAS)	*P* < 0.05

Sun and Yu [25] (2000)	Liver	80	40	AT	Morphine 30 mg qn	Ashi point	12 h qn n.r.	Response rate (duration of pain relief more than 3 h)	*P* < 0.05

Han and Chen [26] (2012)	Liver	25	25	Wrist-ankle AT	Drug (three-step analgesic ladder)	Ashi point	10–12 h qd 10 days	Response rate NRS	*P* > 0.05

Dan et al. [27] (1998)	Miscellaneous	37 42	34 34	A: AT B: AT + C	Drug (three-step analgesic ladder) Drug (three-step analgesic ladder)	LI4, SJ6, and PC6 Chest pain: ST40, HT8 Pain in ribs: GB40, LR3 Abdominal pain: SP6, ST36	0.5–1.5 h qd or bid 10 days	Reduction of pain level VRS	*P* < 0.05 *P* < 0.001

Qiao et al. [28] (2008)	Miscellaneous	34	32	AT + C	Drug (three-step analgesic ladder)	Back-shu points, Ashi point, ST36	30 min/qd 14 days	Reduction of pain level NRS plus VRS	*P* < 0.01

Zhang et al. [29] (2000)	Miscellaneous	22	22	AT	Drug (three-step analgesic ladder)	LI4, PC6, Ashi point, and Back-shu points	0.5–1 h bid 20 days	Reduction of pain level VRS	*P* < 0.05

Cui [30] (2013)	Miscellaneous	20	20	AT	Morphine	LI4, PC6, and Ashi point	35 min bid 15 days	Response rate (degree of pain relief more than 60%)	*P* < 0.05

Tan et al. [31] (2012)	Miscellaneous	37 38 31	32 36 33	Mild: AT + C Moderate: AT + C Severe: AT + C	Drug (three-step analgesic ladder) Drug (three-step analgesic ladder) Drug (three-step analgesic ladder)	LI4, PC6, Ashi point, and Back-shu points Lung cancer: LI4, PC6, and LU6 Liver cancer: LI4, PC6, GB34, and LR6 Colon cancer: LI4, PC6, RN12, ST36, and SJ6	0.5–1 h qd 3 weeks	Reduction of pain level VRS	*P* < 0.01 *P* < 0.05 *P* > 0.05

Peng [32] (2009)	Miscellaneous	11	11	EA + AA	Sham EA + sham AA	Based on syndrome differentiation, disease differentiation	30 min qd 21 days	(1) Pain in score (100 mm VAS) (2) QLQ-C30	*P* > 0.05

Dang and Yang [33] (1995)	Stomach	16	16	AT	Drug (three-step analgesic ladder)	ST36, SP6, ST34, PC6, LI11, LI4, and Ashi points Chest pain: CV17, SP21, TE6, and GB34 Low-back pain: GV12, SI11, SI3, and GB39 Selecting 4~5 mean points and 2~4 auxiliary points each time, right and left were alternately treated	20 min qd-tid 2 months	(1) Response rate (2) QOL VRS	*P* > 0.05

Peng et al. [34] (2012)	Miscellaneous	23	24	EA + C	Drug (three-step analgesic ladder)	LI4, PC6, ST36, and SP6 Back-shu points, front-mu points, and xi-cleft points related to pathological viscera	30 min qd 7 days	Response rate (degree of pain relief more than 31%) NRS	*P* > 0.05

Huang and Zhang [35] (2012)	Miscellaneous	43	42	AT + C	Drug (three-step analgesic ladder)	ST36, Ashi point, Back-shu points, and Jiaji points according to symptoms	40 min qd 2 weeks	(1) Reduction of pain level (2) KPS VRS	*P* < 0.05

Bian [36] (1999)	Miscellaneous	32 38	33 33	A: EA B: EA + C	Drug (three-step analgesic ladder) Drug (three-step analgesic ladder)	ST36, PC6, and SJ8 Chest pain: BL13 Gastric pain: BL20, BL21 Abdominal pain: SP6 Low-back pain: BL23	30 min bid	Response rate (duration of pain relief more than 12 h)	*P* > 0.05 *P* < 0.01

Zhang [37] (2014)	Miscellaneous	30	30	AT + C	Drug (three-step analgesic ladder)	Lung cancer: LI4, PC6, LU6, and ST36 Liver cancer: LI4, GB34, LR6, ST36, and LR3 Colon cancer: LI4, PC6, RN12, ST36, and SJ6	30 min qd 7 days	(1) Reduction of pain level (2) QOL NRS	*P* > 0.05

Alimi et al. [38] (2003)	Miscellaneous	29	30	AA	Sham AA (auricular seeds at nonacupoint)	Ear points based on potential difference	Once a month 2 months	Pain in score (100 mm VAS)	*P* < 0.01

T: treatment group, C: control group, AA: auricular-acupuncture, AT: acupuncture, EA: electroacupuncture, n.r.: not reported, NRS: numerical rating scale, VAS: visual analogue scale, and VRS: verbal rating scale.

**Table 2:** Grade Quality of evidence of acupuncture plus drug therapy for cancer pain.

Outcomes	Illustrative comparative risks^*∗*^ (95% CI)		Relative effect (95% CI)	Number of participants (studies)	Quality of the evidence (GRADE)
	Assumed risk Control	Corresponding risk Acupuncture plus analgesic			
Response rate for relieving pain pain scale Follow-up: 7–30 days	772 per 1000 816 per 1000	Study population 911 per 1000 (841 to 980) Moderate 963 per 1000 (889 to 1000)	RR 1.18 (1.09 to 1.27)	845 (11 studies)	⊕ ⊝ ⊝ ⊝ Very low^1,4^

Onset time of pain relief Follow-up: 7–28 days		The mean onset time of pain relief in the intervention groups was 1.06 standard deviations lower (1.34 to 0.79 lower)		230 (4 studies)	⊕ ⊝ ⊝ ⊝ Very low^1,2,3^

Duration of pain relief Follow-up: 7–28 days		The mean duration of pain relief in the intervention groups was 1.03 standard deviations higher (0.49 to 1.57 higher)		268 (5 studies)	⊕ ⊝ ⊝ ⊝ Very low^1,2,3,4^

QOL Follow-up: 7–30 days		The mean score of QOLl in the intervention groups was 1.44 standard deviations higher (0.43 to 2.44 higher)		196 (3 studies)	⊕ ⊝ ⊝ ⊝ Very low^1,2,3,4^

^1^None of the trials were blinded; most of them did not mention randomization process and allocation concealment.

^2^Total sample size is less than calculated optimal information size.

^3^Published evidence is limited to a small number of trials, all of which are showing benefits.

^4^Confidence intervals with minimal overlap, the heterogeneity is significant.

The GRADE profile noted “*∗*” means the basis for the assumed risk (e.g., the median control group risk across studies) is provided in footnotes. The corresponding risk (and its 95% confidence interval) is based on the assumed risk in the comparison group and the relative effect of the intervention (and its 95% CI).
